# Inositol Signaling in the Basidiomycete Fungus *Schizophyllum commune*

**DOI:** 10.3390/jof7060470

**Published:** 2021-06-10

**Authors:** Reyna Murry, Lea Traxler, Jessica Pötschner, Thomas Krüger, Olaf Kniemeyer, Katrin Krause, Erika Kothe

**Affiliations:** 1 Institute of Microbiology, Friedrich Schiller University Jena, Microbial Communication, Neugasse 25, 07743 Jena, Germany; reyna.murry@uni-jena.de (R.M.); lea.traxler@uni-jena.de (L.T.); jessica.poetschner@uni-jena.de (J.P.); katrin.krause@uni-jena.de (K.K.); 2 Leibniz Institute for Natural Product Research and Infection Biology—Hans Knöll Institute, Molecular and Applied Microbiology, Adolf-Reichwein-Straße 23, 07745 Jena, Germany; thomas.krueger@leibniz-hki.de (T.K.); olaf.kniemeyer@leibniz-hki.de (O.K.)

**Keywords:** inositol phosphate signaling, inositol monophosphatase, cell wall integrity, sexual development, intracellular trafficking, heavy metals

## Abstract

Intracellular signaling is conserved in eukaryotes to allow for response to extracellular signals and to regulate development and cellular functions. In fungi, inositol phosphate signaling has been shown to be involved in growth, sexual reproduction, and metabolic adaptation. However, reports on mushroom-forming fungi are lacking so far. In *Schizophyllum commune*, an inositol monophosphatase has been found up-regulated during sexual development. The enzyme is crucial for inositol cycling, where it catalyzes the last step of inositol phosphate metabolism, restoring the inositol pool from the monophosphorylated inositol monophosphate. We overexpressed the gene in this model basidiomycete and verified its involvement in cell wall integrity and intracellular trafficking. Strong phenotypes in mushroom formation and cell metabolism were evidenced by proteome analyses. In addition, altered inositol signaling was shown to be involved in tolerance towards cesium and zinc, and increased metal tolerance towards cadmium, associated with induced expression of kinases and repression of phosphatases within the inositol cycle. The presence of the heavy metals Sr, Cs, Cd, and Zn lowered intracellular calcium levels. We could develop a model integrating inositol signaling in the known signal transduction pathways governed by Ras, G-protein coupled receptors, and cAMP, and elucidate their different roles in development.

## 1. Introduction

*Schizophyllum commune* is a saprotrophic white-rot fungus that can complete its life cycle within two weeks on artificial media. It has a long haploid growth phase, and the full genome of *S. commune* has been published [1]. The basidiomycete is accessible to transformation and genetic modification [2,3,4]. With regard to mating, *S. commune* has been used extensively to study signaling pathways [5,6] involving inositol signaling [7,8,9,10].

Inositol phosphate signaling has been intensively studied since the 1980s [11,12]. In ascomycete and yeast-forming fungi, inositol phosphate signaling plays a major role in a wide range of biological processes, including metabolic adaptation, apoptosis, fungal virulence, vesicle trafficking, and sexual development [13,14,15,16]. The filamentous basidiomycete fungus *S. commune* could be shown to include phosphate signaling in its life cycle. The first evidence for inositol and Ras signaling cross-talk was suggested to be in phosphate storage [8,10].

The tetrapolar mating system of basidiomycetes is controlled by two non-linked loci. The *A* locus encoding several pairs of homeodomain transcription factors with multiple mating specificities in each allele, and the *B* loci with a pheromone receptor system allow for the recognition of multiple non-self-specificity pheromones with each of the two pheromone receptors. This elaborate mating system controls the formation of the dikaryon that is capable of forming fruiting bodies in response to extracellular signals under natural growth conditions. Since mushroom development is of importance for food production and ecosystem functions, the investigation of signal perception and transduction with its integration into sexual development is of broad interest (see [1]).

**Figure 1:**
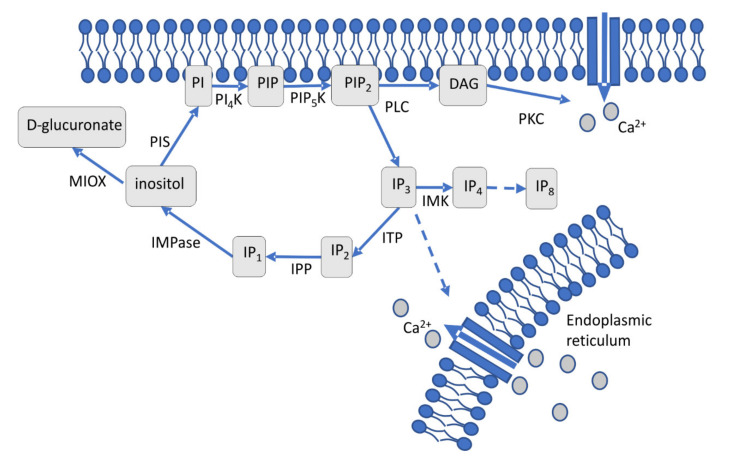
Schematic representation of cellular inositol signaling. Phosphatidylinositol specific phospholipase C (PLC) cleaves phosphatidylinositol (PI)-4,5-bisphosphate (PIP_2_) generating two second messengers, diacylglycerol (DAG) and inositol 1,4,5 triphosphate (IP_3_). IP_3_ can be diverted into higher phosphorylated IP_4_ through IP_8_ or dephosphorylated to inositol through IP_2_ and IP_1_. Inositol, regenerated by IMPase from IP_1_, is the precursor of membrane-bound PIP_2_.

An extracellular stimulus that activates a membrane-associated phospholipase C (PLC) leads to cleavage of phosphatidylinositol-4,5-bisphosphate (PIP2) into diacylglycerol (DAG), which stays at the membrane, and soluble inositol 1,4,5-triphosphate IP_3_ (Figure 1) [17,18]. Through DAG, protein kinase activation leads to Ca^2+^ channel opening and subsequent calcium influx [19]. IP_3_ is involved in calcium release from the endoplasmic reticulum [20], phosphate storage via polyphosphorylated inositol phosphates (up to IP_7_ and IP_8_) [10,21,22] and connected to other signaling cascades, including Ras signaling [8,10,23]. The cycle is restored through IP_2_ and IP_1_, which are dephosphorylated to inositol by the key enzyme, inositol monophosphatase (IMPase). This enzyme is specifically inhibited by lithium, which leads to lower inositol and calcium levels in the cell [24]. The sugar inositol can enter catabolic pathways via inositol oxygenase activity forming glucuronic acid [25]. Inositol monophosphate (IP_1_) and inositol monophosphatase (IMPase), thus, are key players in a highly complex network of signaling pathways.

The enzyme IMPase is quite well studied and known to require magnesium and to be non-competitively inhibited by lithium [26]. Therefore, in humans, the enzyme is addressed in the therapy of manic-depressive disorder with lithium-based drugs [27]. In euryhaline eel, IMPase plays a role in the adaptation to hypertonic conditions [28], while in plants, it contributes to drought and salt resistance, as well as response to pathogens and symbionts [29]. In ascomycetes, yeasts and the filamentous fungus *Podospora anserina* have been studied [16]. IMPase is highly conserved among basidiomycetes, including the yeast-forming *Cryptococcus neoformans* [13]. To understand the role in *S. commune*, we used overexpression of IMPase and discovered potential roles in cell wall integrity and cellular trafficking. Since it has been reported that inositol signaling is also impaired under metal stress [30], we were interested in inositol signaling in basidiomycete fungi, using the model organism *S. commune*. For this purpose, IMPase overexpression mutants were compared with wildtypes for their metal tolerance against Sr, Cs, Cd, and Zn. Regulation of the genes belonging to inositol signaling was assessed with respect to metal stress using microarray and proteome analyses [31].

## 2. Materials and Methods

### 2.1. Phylogenetic Analysis and Chromosomal Organization of imp1

To construct a phylogenetic tree, the genome sequence [1] was used for sequence searches at https://mycocosm.jgi.doe.gov and https://www.ncbi.nlm.nih.gov, visualized using Launch Jalview Desktop (http://www.jalview.org/; all accessed on 24 October 2018). Tree calculation with MrBayes was carried out via the online tool CIPRES Science Gateway and visualized using FigTree v1.4.3. If necessary, tree captions and information were edited using Adobe Illustrator.

The chromosomal organization based on predicted genes, size, and functional data from the genome sequence was displayed with ChromoMapper.

### 2.2. Fungal Growth and Microscopy

*S. commune* (strains listed in Supplementary Table S1) was used to construct *imp1* overexpressing strains OEIMP4 and OEIMP6 (primers, Supplementary Table S2; for construction, Supplementary Figure S1) and an empty vector control EVC. The gene *imp1* under control of the constitutive, strong *tef1* promotor was cloned using yeast recombination of amplified sequences (Q5 High-Fidelity polymerase, New England Biolabs, Ipswich, USA) using primers with 3′ and 5′ overlap (Supplementary Table S2). Shuttle plasmid pRS415 was linearized with *Sac*I and *Sac*II, the *Hind*III/*Pci*I fragment (2.9 kb) ligated into pSKtrp resulting in pOEIMP. Circular plasmid pOEIMP was introduced in *S. commune* T33 according to [32]. Transformants were screened by PCR using primers 5F and 5R (see Supplementary Table S2).

Strains were cultivated on CYM [33] complemented with 4 g/L tryptophan if needed. Calcofluor white (0, 125, 250, 500 µg/mL), Congo red (0, 125, 250, 500 µg/mL) and SDS (0, 20, 60, 100 µg/mL) were added for respective analyses for 10 days. Growth was checked measuring the mycelium diameter every day and significant differences between treated and control cultures were confirmed by Student’s *t*-test (*p*-value ≤ 0.05).

To visualize cellular trafficking, SynaptoRed FM 4-64 (3.2 µM for 40 min, washed twice with PBS: 137 mM NaCl, 2.7 mM KCl, 10 mM Na_2_HPO_4_, 1.8 mM KH_2_PO_4_, pH 7.4) was used after 3 days of cultivation in 24 well plates (Greiner Bio One, Frickenhausen, Germany) at room temperature with 150 µL agar and 150 µL liquid medium to keep the culture moist. The sample was directly observed (CLSM Zeiss LSM 780, Jena, Germany; 40×/1.30 NA EC Plan-Neofluar objective and transmission light or fluorescence Ar/ML 488/514 nm laser with 0.2% intensity). The signal from 622 nm to 759 nm was detected with GaAsP detector. Pinhole size and laser dwell time were minimized. Finalization and intensity measurements of images were carried out with the program Zen2012 (Zeiss, Jena, Germany).

For growth of *S. commune* with metal stress, monokaryotic *S. commune* 12-43 (*ura*^−^) and S. commune T33 (*trp*^−^*, ura*^−^), the empty vector control and the inositol monophosphatase overexpressing *S. commune* OEIMP4 were cultivated on minimal medium (MM + ura + trp: 20 g/L glucose, 2 g/L aspartic acid, 1 g/L K_2_HPO_4_, 0.5 g/L KH_2_PO_4_, 0.5 g/L MgSO4, 60 μg/L thiamine hydrochloride, 11.2 mg uracil, 1 g tryptophan, 16 g/L agar; pH 6) [34] at 28 °C for 14 days. Metal salt solutions of SrCl_2_, CsCl, ZnCl_2_ or CdCl_2_ were added to the medium after autoclaving. Growth rates were calculated from the mycelial diameter in two dimensions, with all experiments in triplicates and significant differences confirmed by *t*-test (*p*-value ≤ 0.05).

### 2.3. Overexpression of imp1

RNA isolation was performed based on manufacturer’s protocol (RNeasy Plant Mini Kit, Qiagen, Hilden, Germany). A total of 500 ng RNA were transcribed to cDNA (QuantiTect Reverse Transcription, Qiagen, Hilden, Germany). All experimental cDNA samples were assayed in triplicates. Expression of *imp1* was normalized using *tef1* as a reference after qRT-PCR (smart Cycler II and 25 μL Cepheid reaction tubes, Cepheid, Sunnyval, USA). The qPCR mix consisted of 12.5 μL Maxima SYBR Green/ROX qPCR Master mix (Thermo Fisher Scientific, Hamburg, Germany), 0.5 μL of 10 pmol/μL of each forward and reverse primers (see Table S2), 9.5 μL DNAse, RNAse, pyrogenase free water (Carl Roth, Karlsruhe, Germany), and 2 μL of 25 ng/μL cDNA. The cycling program contained a gradient: 95 °C for 120 s, 40 cycles of 95 °C for 20 s, 60 °C for 20 s, and 72 °C for 15 s; 40 cycles of 60–90 °C for 0.2 °C/s.

### 2.4. Calcium Levels under Metal Stress

*S. commune* 12-43 was grown on MM + ura with either 100 mM SrCl_2_, 75 mM CsCl, 10 mM ZnCl_2_, or 0.5 mM CdCl_2_ topped with a cellophane foil. After 14 days at 28 °C, the mycelium was harvested with a plastic spatula and dried for 18 h at 70 °C until constant weight and the mycelium homogenized in an agate mortar. The experiment was performed in five replicates. Ca as well as metal contents were measured in a mixed sample of five biological replicates after microwave extraction using ICP-MS (Inductively Coupled Plasma - Mass Spectrometry, XSeries II, Thermo Fisher Scientific, Bremen, Germany; for details compare [35]).

### 2.5. Proteome Study

Empty vector control (EVC1) and *imp1* overexpressing *S. commune* OEIMP4 were incubated as before and proteins, enriched for membrane associated proteins, were extracted (ReadyPrep Protein Extraction Kit Signal, Bio-Rad, Munich, Germany) based on manufacturer’s protocol after grinding mycelia with liquid nitrogen. A sonication step (VCX 130 PB, Sonics and Material, Newtown, CT, USA) was performed, and concentrations of isolated proteins were determined (Sigma-Aldrich, Darmstadt, Germany) [36]. To purify the protein, 50 µM protein were loaded onto 10% polyacrylamide gels. After electrophoresis, gels were washed two times with ultrapure water, protein bands were excised and cut into cubes of approx. 1 mm^3^ and transferred to 1.5 mL tubes. In-gel digestion was performed [37] using 50 ng/µL trypsin-LysC (mass spec grade, Promega, Mannheim, Germany) in 50 mM NH_4_HCO_3_. Peptides were extracted with trifluoroacetic acid (TFA) and increasing concentrations of acetonitrile (50, 70, and 90%), purified with 10 kDa MWCO filters (VWR, Langenfeld, Germany), and dried peptides solubilized in MS buffer (0.05% TFA in 2% acetonitrile–98% H_2_O) before being subjected to LC-MS/MS analysis (Ultimate 3000 nano RSLC, QExactive HF, Thermo Fisher Scientific, Waltham, MA, USA). Initial peptide trapping for 5 min on an Acclaim Pep Map 100 column (2 cm × 75 µm, 3 µm) at 5 µL/min was followed by separation on an analytical Acclaim Pep Map RSLC nano column (50 cm × 75 µm, 2 µm). Mobile phase gradient elution of eluent A (0.1% *v/v* formic acid in water) mixed with eluent B (0.1% *v/v* formic acid in 90/10 acetonitrile/water) was performed (buffer B for 0 min 4%, 5 min 5%, 15 min 6%, 100 min 8%, 150 min 12%, 250 min 23%, 300 min 34%, 320 min 41%, 340 min 52%, 350 min 60%, 360 min 75%, 365–375 min 96%, 375.1–400 min 4%). Positively charged ions were generated at a spray voltage of 2.2 kV using a stainless-steel emitter attached to the Nanospray Flex Ion Source (Thermo Fisher Scientific, Waltham, USA). The quadrupole/orbitrap instrument was operated in Full MS/data-dependent MS2 (top10) mode. Precursor ions were monitored at m/z 300–1500 at a resolution of 120k FWHM using a maximum injection time of 100 msec and an automatic gain control target of 1e6. HCD fragmentation at 30% normalized collision energy generated MS2 ions, which were scanned at 15 k FWHM (ITmax = 100 ms, AGC = 2∙10^5^). Dynamic exclusion of precursor ions was set to 35 s. The LC-MS/MS instrument was controlled by Chromeleon 7.2, QExactive HF Tune 2.8, and Xcalibur 4.0 software (Thermo Fisher Scientific, Waltham, MA, USA).

The obtained tandem mass spectra were searched against the JGI database of *S. commune* H4-8 v3.0 (https://mycocosm.jgi.doe.gov/Schco3/Schco3.home.html; accessed on 2 February 2017) using Proteome Discoverer PD 2.1 and the algorithms of Mascot 2.4, Sequest HT, and MS Amanda. Two missed cleavages were allowed for trypsin digestion. The precursor mass tolerance was set to 10 ppm and the fragment mass tolerance was set to 0.02 Da. Modifications were defined as dynamic Met oxidation and static Cys carbamidomethylation. At least two peptides per protein and a strict false discovery rate < 1% (reverse decoy) were required. Label-free quantification of the Top 3 unique peptides per protein was based on the precursor ion area detector approach implemented in PD 2.1. For quantification, data were normalized using the total peptide amount approach. The mass spectrometry proteomics data have been deposited to the ProteomeXchange Consortium via the PRIDE [38] partner repository with the dataset identifier PXD026465.

### 2.6. Transcriptome Analyses

An mRNA sequencing approach was analyzed and enzymes of inositol signaling were found to be significantly regulated under metal stress [39]. The two significantly regulated genes, myo-inositol oxygenase (XM_003027475.1) and a hypothetical inositol polyphosphate phosphatase (XM_003036036.1) were selected to confirm regulation under metal stress using qPCR.

RNA was extracted from mycelium of *S. commune* 12-43 grown with 75 mM SrCl_2_, 50 mM CsCl, 5 mM ZnCl_2_, or 0.2 mM CdCl_2_ (RNeasy Plant Mini Kit, Qiagen, Hilden, Germany). From 500 ng RNA, cDNA was synthesized (QuantiTect Reverse Transcription Kit, Qiagen, Hilden, Germany). The qRT-PCR was performed with the qTOWER3 from AnalytikJena (Jena, Germany). Each reaction consisted of 3.125 µL Maxima SYBR Green/ROX qPCR Mastermix (2x; ThermoFischer Scientific, Waltham, USA), 0.5 µL each of 10 pmol/µL primers, see Supplementary Table S2), 1.125 µL water and 1 µL cDNA 1:10 diluted. The qPCR was run with 10 min initial denaturation at 94 °C, 40 cycles of 20 s 94 °C, 20 s 59 °C, and 20 s 72 °C followed by 2 min final elongation. A melting curve was measured after every run from 60 to 94 °C with ∆1 °C per 30 s. All measurements were done in three biological and three technical replicates, with each a negative control and a “no reverse transcriptase” control. Relative expression was calculated using the genes coding for actin 1 (XM_003026104.1), ubiquitin (XM_003036409.1) and translation elongation factor1α (XM_003037215.1) [40]. Significance was tested with a two-tailored *t*-test with unequal variances and a *p*-value of ≤0.05.

Microarray data (GEO omnibus acc. no. GSE172373) were evaluated for regulation of enzymes involved in inositol signaling. In the microarray, the gene regulation of *S. commune* 12-43 grown on complex yeast medium [33] was compared with growth in presence of multi-heavy metal containing seepage water and to co-isogenic *S. commune* W22 grown with 0.01 mM cadmium nitrate. All enzymes of inositol signaling that were regulated under metal stress were selected from this microarray data.

## 3. Results

### 3.1. A Basidiomycete Clade of IMPases

**Figure 2:**
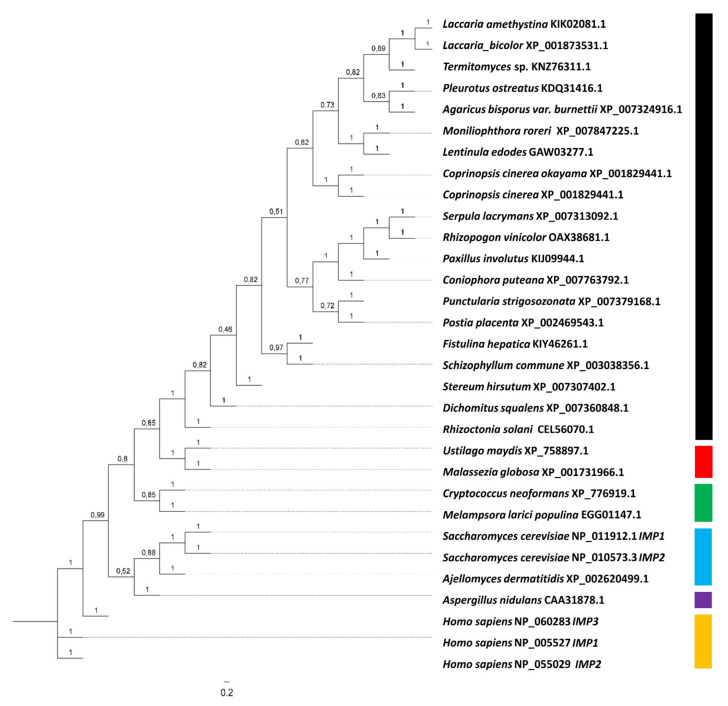
Phylogenetic tree for IMPases. Accession numbers (GeneBank) are given for all sequences. IMPase clades are indicated with filamentous Basidiomycota (black), yeast-like Basidiomycota I (red), yeast-like Basidiomycota II (green), yeast-like Ascomycota (blue), filamentous Ascomycota (purple) und human IMPases (yellow).

The gene coding for IMPase, *imp1,* is located in scaffold 2 of the genome of *S. commune* H4-8 and has a coding region of 1247 bp interrupted by five introns. IMPase is highly conserved among basidiomycetes, and the single gene *imp1* in *S. commune* encodes 340 aa containing all three motifs for substrate and metal binding, and nucleophilic activation. Alignments and phylogenetic analysis including human, yeast, and basidiomycete sequences revealed the existence of different evolutionary clades in IMPases (Figure 2). The highest aa identity was shared between the *S. commune*, *Laccaria amethystina* (67%), *Laccaria bicolor* (67%), *Termitomyces* (66.5%), and *Fistulina hepatica* (64%) sequences, all of basidiomycete origin.

Surrounding genes included a conserved zinc finger transcription factor and a chaperone of the DnaJ superfamily. This direct neighborhood is conserved with a zinc finger protein, *zfand1,* and also with the human gene coding for inositol monophosphatase 1.

### 3.2. Imp1 Overexpression Leads to Improved Cell Wall Integrity

**Figure 3:**
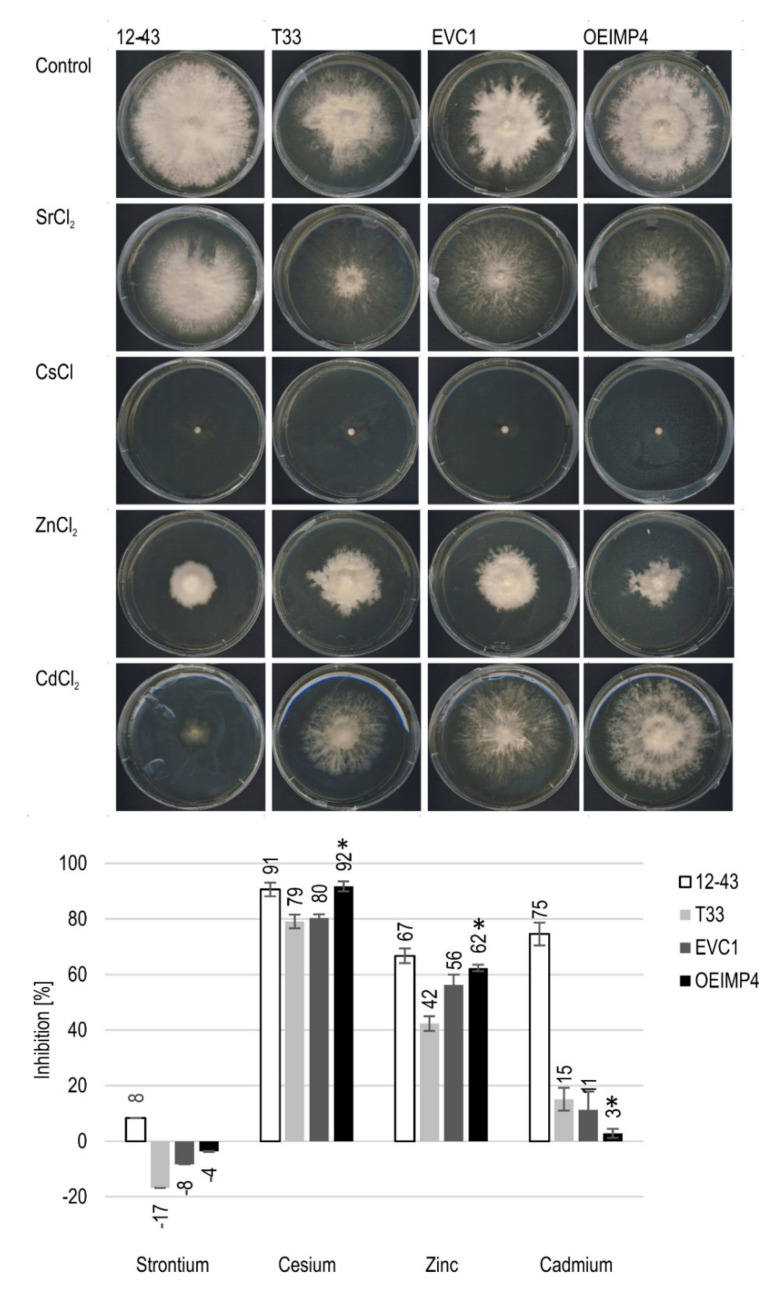
Growth inhibition of metals. Growth morphology (**top**) and inhibition calculated from mycelia diameter (**bottom**) of *S. commune* 12-43, T33, EVC1 and OEIMP4 in three biological replicates.

Overexpression was achieved with the gene *imp1* under the control of the strong promotor of *tef1*, originally leading to constitutive expression of translation elongation factor EF1α. The overexpression was verified by RT-qPCR (Supplementary Figure S2). A remarkable increase of 42- and 67-fold up-regulation was confirmed for the two independent transformants, OEIMP4 and OEIMP6, respectively. Growth was only slightly retarded upon long term incubation for *imp1* overexpressing strains as compared to a wildtype and the empty vector control without obvious changes in morphology of mycelia (Figure 3).

A more detailed analysis of cellular effects of overexpression revealed a role in cell wall integrity, as decreased susceptibility against calcofluor white, binding to chitin, Congo red for specific polysaccharide staining, and SDS as membrane-active detergent were observed (Supplementary Figure S4). The results indicate that Imp1 and inositol signaling might play a role in biosynthesis and organization of the cell wall of *S. commune*. Since stability against cell wall stress was observed, growth with heavy metals exerting such stress was screened.

### 3.3. Influence of Altered Inositol Signaling on Metal Tolerance

The influence of altered inositol signals on metal tolerance was investigated by exposing IMPase-overexpressing *S. commune* OEIMP4 to heavy metal stress. For all four tested metals, the strain *S. commune* 12-43 performed inferior as compared to *S. commune* T33 and its derivatives, *S. commune* EVC1 and *S. commune* OEIMP4 (Figure 3). In addition to higher inhibition through metals, *S. commune* 12-43 formed more aerial mycelium. The remaining three strains all showed, unexpectedly, improved growth with Sr. However, this is due to the fact that aerial mycelium was reduced in favor of more (and thus faster) substrate hyphae formation. The inositol monophosphatase overexpression led to a significantly higher inhibition by Cs and Zn, which was not seen with Cd.

The intracellular calcium levels were distinctly reduced in *S. commune* 12-43 grown in the presence of heavy metals. The difference was most evident in the presence of Sr, but clearly visible also for Cs and Cd, where calcium levels were reduced (Table 1).

### 3.4. Inositol Signaling Related Gene Regulation in Metal Stress

**Figure 4:**
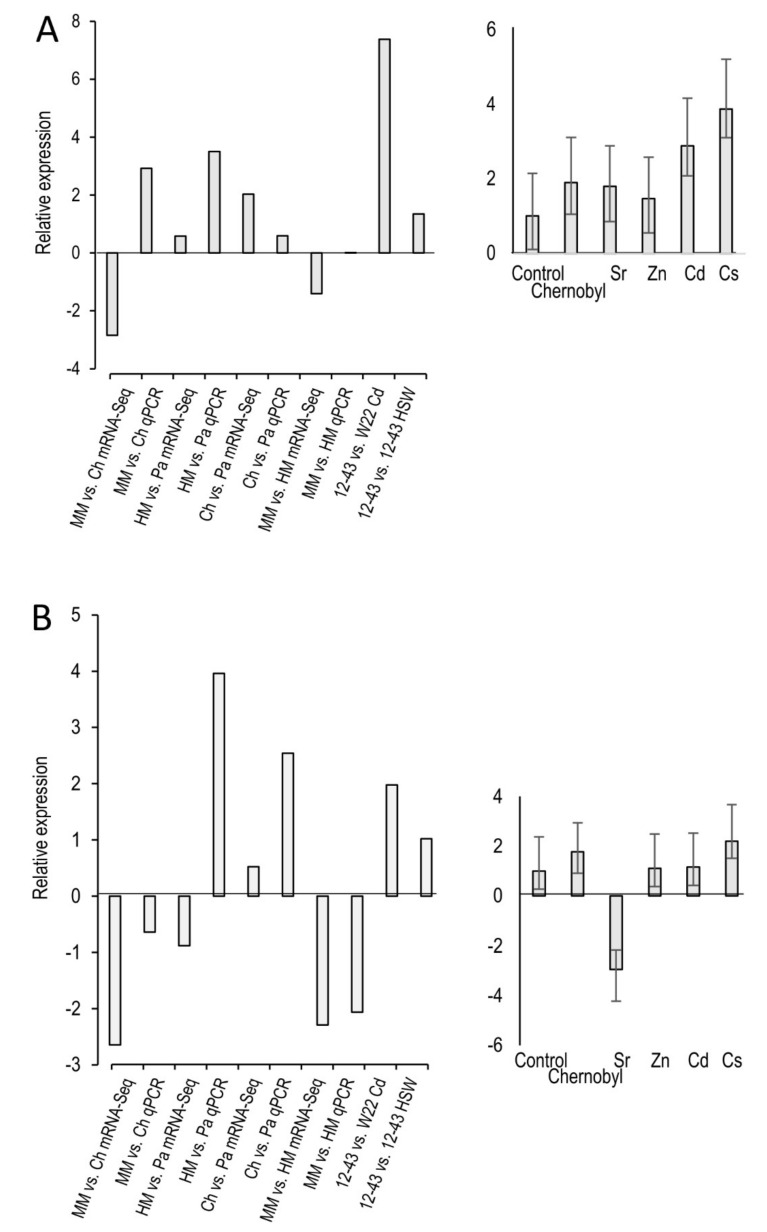
Validation of mRNA sequencing by microarray and qPCR (left) and further investigation by qPCR (right) for inositol polyphosphate phosphatase (**A**) and myo-inositol monooxygenase (**B**). *S. commune* 12-43 or *S. commune* W22 were grown on minimal medium (MM), minimal medium containing the heavy metals present in Paradies park soil (HM), directly in Chernobyl soil (Ch), Paradies park soil (PA) or seepage water (HSW).

The general direction of the gene regulation of the two genes of interest could be confirmed using qPCR. Exceptions were the comparisons of minimal medium versus Chernobyl soil for the hypothetical inositol polyphosphate phosphatase gene and heavy metal medium versus Paradies park soil for the *myo*-inositol oxygenase gene (Figure 4). The two microarray comparisons (Supplementary Table S3) showed that increased metal stress lead to a higher expression of both genes. This also tended to be the case for the first three comparisons of the mRNA sequencing, but not for the comparison minimal medium versus heavy metal medium. The qPCR investigation confirmed that the hypothetical polyphosphate phosphatase gene was highly up-regulated under the influence of all metals tested. The Myo-inositol oxygenase gene just tended to be up-regulated under metal stress. However, in the presence of zinc and cadmium, no regulation could be recognized and clear down-regulation in the case of strontium.

The two comparisons of the microarray data mostly reacted under metal stress with gene regulation in the same direction for the same genes. A difference was most likely to be seen in the regulation of protein kinase C, which occurs outside of the immediate inositol cycle (see Figure 1). It could also be seen that under metal stress, kinases tended to be up and phosphatase tended to be down-regulated (Table S3). Down-regulation was clearly observed for inositol triphosphate phosphatase, which catalyzes the phosphorylation of inositol triphosphate to inositol diphosphate.

### 3.5. Imp1 Overexpression and Changed Intracellular Trafficking

**Figure 5:**
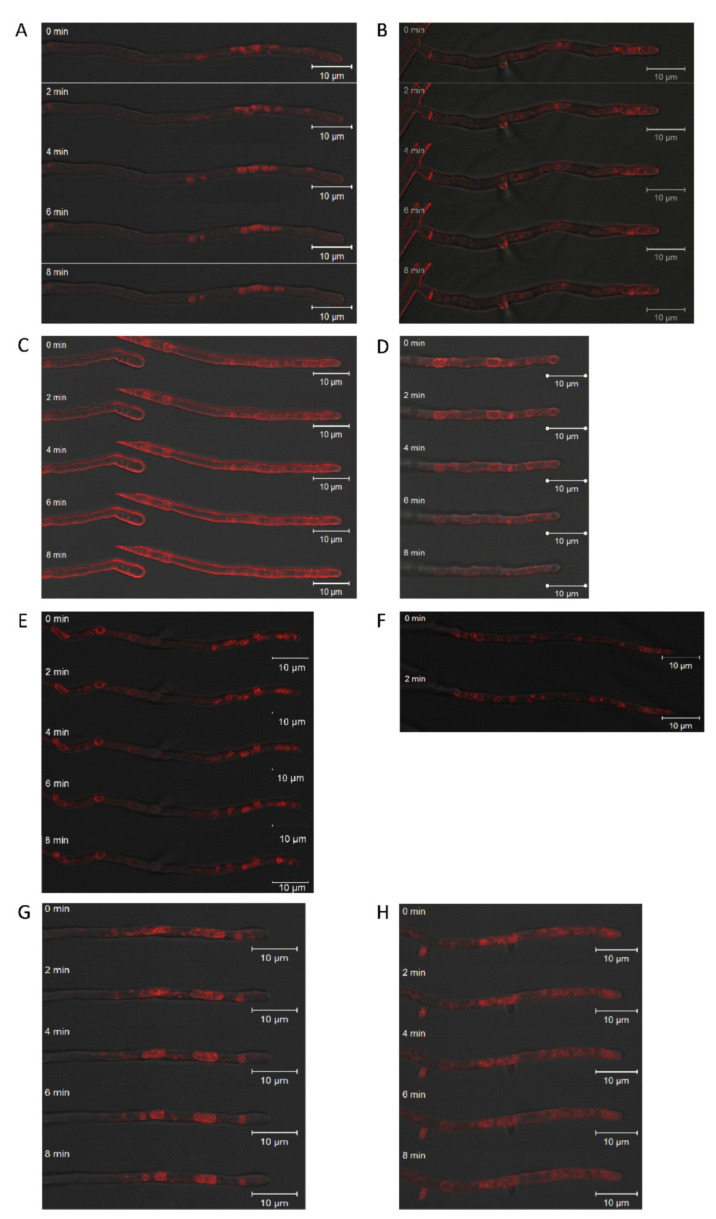
Effect of brefeldin A on intracellular and membrane trafficking. *S. commune* T33 (**A**,**B**), the empty vector control EVC1 (**C**,**D**), and overexpression transformants OEIMP4 and OEIMP6 (**E**–**H**) without (**A**,**C**,**E**,**G**) and with 5 µm brefeldin (**B**,**D**,**F**,**H**) were compared. Membranes were stained using FM4-64 and monitored with time-lapse mode for 0–8 min.

Fungal endocytosis and exocytosis are key processes in growth and cell wall synthesis at the growing tip. SynaptoRed FM 4-64 staining of vesicles and vacuoles in the hyphal apex showed altered vesicle size (Figure 5). In addition, overexpression of *imp1* in conjunction with Brefeldin A treatment, which is a potent inhibitor of exocytosis, increased size and variance in organelles, supporting the notion of involvement of Imp1 in vesicle trafficking. Wavy hyphae in the treated wildtype and *imp1* overexpressing strains show that directional growth might be co-afflicted. Since a strong phenotype thus could be assigned to *imp1* overexpression, changes in proteome were investigated to identify proteins with changed expression profiles in the transformant OEIMP4 as compared to EVC1.

### 3.6. Proteome Analysis Verifies a Function of imp1 in Cellular Trafficking

KOG classification of the 287 proteins with higher abundance upon *imp1* overexpression revealed 43 proteins involved in cellular processes and signaling, 41 in information storage and processing, and 58 in cell metabolism (31 were poorly and 114 not well characterized). Of the 127 proteins with lower abundance, 12 were classified into cellular processes and signaling, 7 in the information storage and processing, and 35 in cell metabolism, with 21 poorly and 52 not well characterized (Supplementary Table S4).

**Figure 6:**
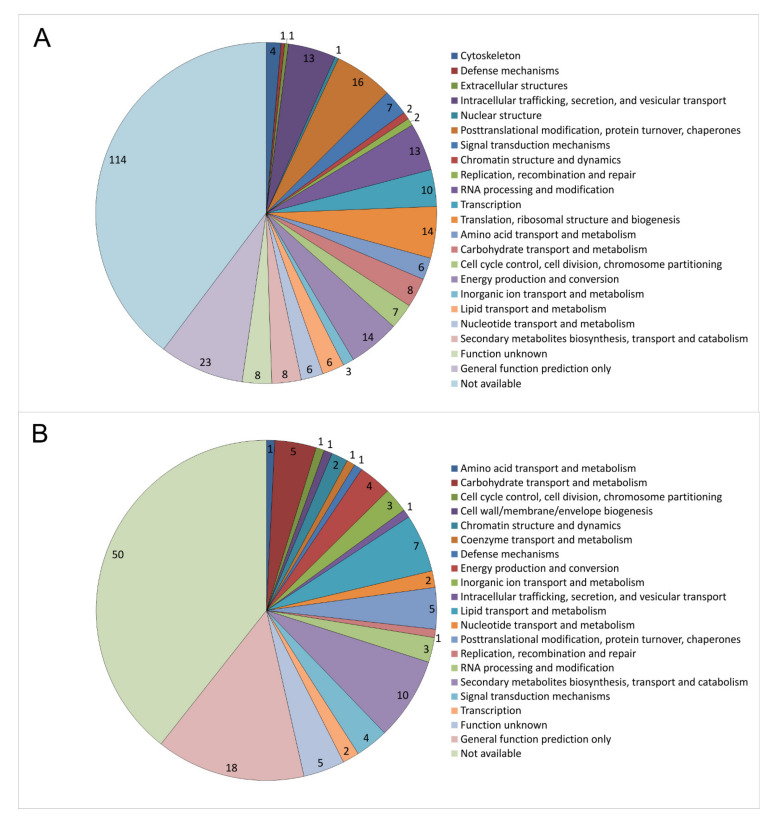
Membrane-associated proteins regulated with respect to *imp1* overexpression in *S. commune* OEIMP4 *versus* empty vector control *S. commune* EVC1. KOG classification of 287 induced (**A**) and 127 repressed proteins (**B**) are given.

The highest regulation in the membrane-associated proteome was recognized with respect to cellular trafficking (Figure 6). Among those were membrane AAA^+^-type ATPase containing the peptidase M41 domain, isopentenyl-diphosphate delta-isomerase involved in the mevalonate pathway, and the antioxidant thioredoxin. Another 14 proteins (signal recognition particle SRP19, ABC transporter, C5 cytosine-specific DNA methylase, acyltransferase, signal recognition particle SRP72, mitochondrial import inner membrane translocase, Tim8 containing zinc finger domain, armadillo type-fold Sec7-like, VPS5/SNX1 containing a PhoX domain, cysteine protease required for autophagy Apg4p/Aut2p, nuclear transport factor 2, t-SNARE, dynamin/VPS1, and two SNF7 proteins) decreased in abundance.

Higher levels were observed for cytoskeleton-associated proteins, three actin binding protein-related proteins and a kinesin-like protein. This supports a function in vesicle trafficking, since vesicles are cargo to the cytoskeleton and moved by motor proteins on the cytoskeletal tracks for directed intracellular movement.

## 4. Discussion

The inositol cycle depends on IMPase activity maintaining a moderate level of the cellular inositol pool [41]. The *imp1* gene encoding IMPase had been found to be up-regulated upon Ras1 activation, suggesting cross-talk between Ras and inositol signaling [8,10]. In addition, lithium as IMPase inhibitor induced a shift to inositol polyphosphates. The phylogenetic analysis obtained now shows conservation between basidiomycete IMPases, including the presence of only one gene for IMPase and linkage to zinc finger (see [42]) and chaperone genes [43]. The conservation is also true for the three strains of *S. commune* with genome sequences available (Supplementary Figure S5).

Overexpression could not only be verified by qPCR, but also independently with the proteome study. A functional role in cell wall structure could be assigned in cell wall integrity and membrane stability, which confirms earlier studies showing particularly amino sugars contents being affected by changes in phosphoinositide in *Neurospora* [44]. Thus, cell wall biosynthesis through formation and membrane fusion of excretory vesicles involved in cell wall biosynthesis during tip growth of fungi is supported with this study.

Downstream of inositol signaling, cellular processes, and signaling related proteins were predominantly affected through *imp1* overexpression, which over-represented intracellular trafficking machinery and cytoskeleton associated proteins. This finding supports the notion that inositol signaling is involved in vesicle trafficking.

The up-regulated signal recognition particle subunits, SRP19 and SRP72, are involved in targeting secretory vesicles in eukaryotes [45,46,47]. Receptor-mediated activation of SRP results in signal peptide cleavage and release of proteins meant for secretion at the endoplasmic reticulum [48,49]. Another protein with elevated abundance, Snf7, is a component of the ESCRT (endosomal sorting complex required for transport)-III complex that is needed for the sorting of proteins into invaginating vesicles of the multivesicular body. The ESCRT complexes are released from the endosomal membrane with the help of an AAA-motif containing ATPase for further rounds of membrane invaginating [50]. Such an ATPase containing an M41 peptidase domain, Snf7, co-regulated with an ABC transporter responsible for export/import of a wide range of substrates including ions and macromolecules, was identified.

Heavy metals are omnipresent in the environment and exert toxic effects at higher concentrations involving oxidative stress [51]. In plants, inositol signaling has been shown to be involved in the reaction to oxidative stress. Here, *S. commune* kinases in inositol signaling were shown to be elevated under metal stress, while phosphatases mainly decreased in abundance. This would shift the cycle to more phosphoinositols like PIP_2_ and less myo-inositol. This is in accordance with the finding that H_2_O_2_-induced cell death is associated with decreased myo-inositol amounts in *Arabidopsis thaliana* and brown algae [30,52]. The up-regulated inositol polyphosphate phosphatase has been shown to be involved in response to ROS and abiotic stress in both plants and animals [53,54]. We could show altered inositol signaling to influence metal tolerance in *S. commune*.

Our investigations showed a down-regulation of inositol multikinases under the influence of metals, which probably leads to lower amounts of highly phosphorylated inositol phosphates and pyrophosphates (IP_7_ and IP_8_; compare [23,55].

The calcium levels of *S. commune* were decreased 4- to 7-fold in the presence of different heavy metals. This could be on one hand due to the presence of extra amounts of metals which were taken up by the hyphae instead of calcium by calcium channels [56] or, on the other hand, could be in a relationship with altered inositol signaling and thus calcium as a second messenger [57]. Calcium has a protective role against ROS and supplemental added calcium leads to increased Cd tolerance [58].

As for vesicle transport, t-SNARE and Sec7-like proteins were induced upon *imp1* overexpression. SEC-7 is involved in vesicular budding and intracellular trafficking between compartments of the Golgi apparatus, and target membrane bound SNARE (t-SNARE) plays an important role in facilitating vesicle fusion in cargo trafficking [59]. For cargo transport, the cytoskeleton is needed that was up-regulated at the proteome level as well.

Long-distance transport along microtubules is suggested by the up-regulation of kinesin, while a plus-end directed microtubule-dependent motor protein [60], dynamin, connects the long-distance and short-distance transport, e.g., at Golgi compartments. Here, again the activity of small GTPases is involved [61,62]. Filamentous actin is organized by the activity of actin-bundling proteins, two of which were detected among the proteins with increased abundances under *imp1* overexpression. Finally, actin-binding proteins and cofilin/tropomyosin permit transport of cargo along actin filaments. This is connected to exocytosis, where vesicles are brought to the vesicle supply center at the growing tip before fusing with the cell membrane to release cell wall components needed for tip growth. The role of actin transport in endocytosis in older hyphal compartments has been discussed controversially for fungi, but seems likely.

**Figure 7:**
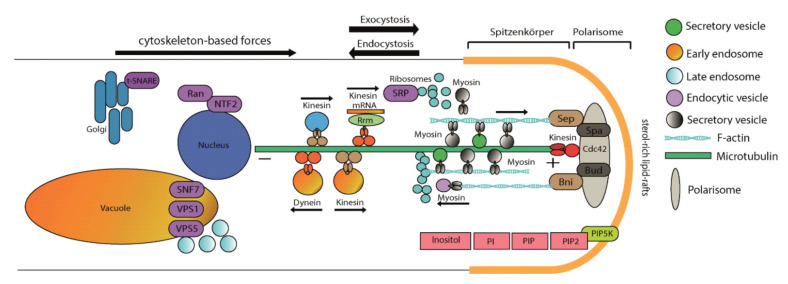
Model integrating inositol signaling in intracellular and membrane trafficking.

To further investigate the role of inositol signaling in intracellular trafficking, we further examined the effect of the strong protein secretion inhibitor brefeldin A in *imp1* overexpressing transformants [63]. Indeed, larger vacuoles were more abundant in the overexpressing strains, supporting the view that vesicle fusion is increased through IMPase activation, and hence the result of inositol signaling. With this study, the view on signal transduction could be expanded (Figure 7) to include inositol phosphate signaling in mushroom forming basidiomycetes.

## Figures and Tables

**Table 1:** Calcium concentrations in *S. commune* 12-43 grown under metal stress.

	Control	100 mM SrCl_2_	75 mM CsCl	10 mM ZnCl_2_	0.5 mM CdCl_2_
Ca [µg/g]	1626 ± 25	219.83 ± 0.02	282 ± 2	234 ± 6	393 ± 2

## Data Availability

All data are publicly available.

## Supplementary Materials

The following are available online at https://www.mdpi.com/article/10.3390/jof7060470/s1, Supplementary Table S1: *S. commune* strains used in this study, Supplementary Table S2: Primers used in this study, Supplementary Figure S1: Plasmid construction for generation of *imp1* overexpression strains, Supplementary Figure S2: Verification of *imp1* overexpression by RT-qPCR, Supplementary Figure S3: Effect of *imp1* overexpression on colony morphology (top) and growth (below) of *S. commune*, Supplementary Figure S4: Susceptibility towards calcofluor white (A), SDS (B), and Congo red (C), Supplementary Table S3: Regulation of genes involved in the inositol signaling cycle, Supplementary Table S4: Intracellular trafficking associated proteins regulated in *imp1* overexpressing transformant pOEIMP4 vs. EVC, Supplementary Figure S5: Chromosomal map of *imp1* surrounding genes in three different strains *S. commune*, H4-8, LoeD, and TatD.

## References

[1] Ohm R.A., de Jong J.F., Lugones L.G., Aerts A., Kothe E., Stajich J.E., de Vries R.P., Record E., Levasseur A., Baker S.E. (2010). Genome sequence of the model mushroom *Schizophyllum commune*. Nat. Biotechnol..

[2] Munoz-Rivas A., Specht C.A., Drummond B.J., Froeliger E., Novotny C.P., Ullrich R.C. (1986). Transformation of the basidiomycete, *Schizophyllum commune*. Mol. Gen. Genet..

[3] Schubert D., Raudaskoski M., Knabe N., Kothe E. (2006). Ras GTPase-Activating protein Gap1 of the homobasidiomycete *Schizophyllum commune* regulates hyphal growth orientation and sexual development. Eukaryot. Cell.

[4] De Jong J.F., Deelstra H.J., Wösten H.A.B., Lugones L.G. (2006). RNA-mediated gene silencing in monokaryons and dikaryons of *Schizophyllum commune*. Appl. Env. Microbiol..

[5] Raudaskoski M., Kothe E. (2010). Basidiomycete mating type genes and pheromone signaling. Eukaryot. Cell.

[6] Jung E.M., Kothe E., Raudaskoski M. (2018). The making of a mushroom: Mitosis, nuclear migration and the actin network. Fungal Genet. Biol..

[7] Wirth S., Kunert M., Ahrens L.M., Krause K., Broska S., Paetz C., Kniemeyer O., Jung E.M., Boland W., Kothe E. (2018). The regulator of G-protein signalling Thn1 links pheromone response to volatile production in *Schizophyllum commune*. Environ. Microbiol..

[8] Knabe N., Jung E.M., Freihorst D., Hennicke F., Horton J.S., Kothe E. (2013). A central role for Ras1 in morphogenesis of the basidiomycete *Schizophyllum commune*. Eukaryot. Cell.

[9] Freihorst D., Brunsch M., Wirth S., Krause K., Kniemeyer O., Linde J., Kunert M., Boland W., Kothe E. (2018). Smelling the difference: Transcriptome, proteome and volatilome changes after mating. Fungal Genet. Biol..

[10] Murry R., Kniemeyer O., Krause K., Saiardi A., Kothe E. (2019). Crosstalk between Ras and inositol phosphate signaling revealed by lithium action on inositol monophosphatase in *Schizophyllum commune*. Adv. Biol. Regul..

[11] Berridge M.J., Irvine R.F. (1989). Inositol phosphates and cell signalling. Nature.

[12] Saiardi A. (2017). Has inositol played any role in the origin of life?. Life.

[13] Lev S., Li C., Desmarini D., Saiardi A., Fewings N.L., Schibeci S.D., Sharma R., Sorrell T.C., Djordjevic J.T. (2015). Fungal inositol pyrophosphate IP_7_ is crucial for metabolic adaptation to the host environment and pathogenicity. MBio.

[14] Li C., Lev S., Saiardi A., Desmarini D., Sorrell T.C., Djordjevic J.T. (2016). Inositol polyphosphate kinases, fungal virulence and drug discovery. J. Fungi.

[15] Li C., Lev S., Saiardi A., Desmarini D., Sorrell T.C., Djordjevic J.T. (2016). Identification of a major IP_5_ kinase in *Cryptococcus neoformans* confirms that PP-IP_5_/IP_7_, not IP_6_, is essential for virulence. Sci. Rep..

[16] Xie N., Ruprich-Robert G., Chapeland-Leclerc F., Coppin E., Lalucque H., Brun S., Debuchy R., Silar P. (2017). Inositol-phosphate signaling as mediator for growth and sexual reproduction in *Podospora anserina*. Dev. Biol..

[17] Berridge M.J., Irvine R.F. (1984). Inositol trisphosphate, a novel second messenger in cellular signal transduction. Nature.

[18] Gillaspy G.E. (2011). The cellular language of myo-inositol signaling. New Phytol..

[19] Nishizuka Y. (1988). The molecular heterogeneity of protein kinase C and its implications for cellular regulation. Nature.

[20] Berridge M.J. (1993). Inositol trisphosphate and calcium signalling. Nature.

[21] Shears S.B. (2017). Intimate connections: Inositol pyrophosphates at the interface of metabolic regulation and cell signaling. J. Cell Physiol..

[22] Wilson M.S., Livermore T.M., Saiardi A. (2013). Inositol pyrophosphates: Between signalling and metabolism. Biochem J..

[23] Saiardi A. (2012). How inositol pyrophosphates control cellular phosphate homeostasis?. Adv. Biol. Regul..

[24] Teo R., King J., Dalton E., Ryves J., Williams R.S., Harwood A.J. (2009). PtdIns(3,4,5)P(3) and inositol depletion as a cellular target of mood stabilizers. Biochem. Soc. Trans..

[25] Bollinger J.M., Diao Y., Matthews M.L., Xing G., Krebs C. (2009). Myo-inositol oxygenase: A radical new pathway for O_2_ and C-H activation at a nonheme diiron cluster. Dalton Transact..

[26] Hallcher L.M., Sherman W.R. (1980). The effects of lithium ion and other agents on the activity of myo-inositol-1-phosphatase from bovine brain. J. Biol. Chem..

[27] Berridge M.J., Downes C.P., Hanley M.R. (1989). Neural and developmental actions of lithium: A unifying hypothesis. Cell.

[28] Kalujnaia S., McVee J., Kasciukovic T., Stewart A.J., Cramb G. (2010). A role for inositol monophosphatase 1 (IMPA1) in salinity adaptation in the euryhaline eel (*Anguilla anguilla)*. FASEB J..

[29] Jia Q., Kong D., Li Q., Sun S., Song J., Zhu Y., Liang K., Ke Q., Lin W., Huang J. (2019). The function of inositol phosphatases in plant tolerance to abiotic stress. Int. J. Mol. Sci..

[30] Ritter A., Dittami S.M., Goulitquer S., Correa J.A., Boyen C., Potin P., Tonon T. (2014). Transcriptomic and metabolomic analysis of copper stress acclimation in *Ectocarpus siliculosus* highlights signaling and tolerance mechanisms in brown algae. BMC Plant Biol..

[31] Erdmann S., Freihorst D., Raudaskoski M., Schmidt-Heck W., Jung E.M., Senftleben D., Kothe E. (2012). Transcriptome and functional analysis of mating in the basidiomycete *Schizophyllum commune*. Eukaryot. Cell.

[32] Van Peer A.F., de Bekker C., Vinck A., Wösten H.A.B., Lugones L.G. (2009). Phleomycin increases transformation efficiency and promotes single integrations in *Schizophyllum commune*. Appl. Environ. Microbiol..

[33] Schwalb M.N., Miles P.G. (1967). Morphogenesis of *Schizophyllum commune.* I. Morphological variation and mating behavior of the thin mutation. Am. J. Bot..

[34] Raper J.R., Hoffman R.M. (1974). Schizophyllum commune. Bacteria, Bacteriophages, and Fungi.

[35] Krauße T., Schütze E., Phieler R., Fürst D., Merten D., Büchel G., Kothe E. (2019). Changes in element availability induced by sterilization in heavy metal contaminated substrates: A comprehensive study. J. Hazard. Mater..

[36] Bradford M.M. (1976). A rapid and sensitive method for the quantitation of microgram quantities of protein utilizing the principle of protein-dye binding. Anal. Biochem..

[37] Shevchenko A., Wilm M., Vorm O., Mann M. (1996). Mass spectrometric sequencing of proteins silver-stained polyacrylamide gels. Anal. Chem..

[38] Perez-Riverol Y., Csordas A., Bai J., Bernal-Llinares M., Hewapathirana S., Kundu D.J., Inuganti A., Griss J., Mayer G., Eisenacher M. (2019). The PRIDE database and related tools and resources in 2019: Improving support for quantification data. Nucleic Acids Res..

[39] Traxler L., Wollenberg A., Steinhauser G., Chyzhevskyi I., Dubchak S., Grossmann S., Günther A., Gupta D.K., Iwannek K.-H., Kirieiev S. (2021). Survival of the basidiomycete *Schizophyllum commune* in soil under hostile environmental conditions in the Chernobyl Exclusion Zone. J. Hazard. Mater..

[40] Pfaffl M.W. (2001). A new mathematical model for relative quantification in real-time RT–PCR. Nucleic Acids Res..

[41] Ferruz N., Tresadern G., Pineda-Lucena A., De Fabritiis G. (2016). Multibody cofactor and substrate molecular recognition in the myo-inositol monophosphatase enzyme. Sci. Rep..

[42] Elsersawi A. (2016). Gene Editing, Epigenetic, Cloning and Therapy.

[43] Cyr D.M., Langer T., Douglas M.G. (1994). DnaJ-like proteins: Molecular chaperones and specific regulators of Hsp70. Trends Biochem. Sci..

[44] Hanson B., Brody S. (1979). Lipid and cell wall changes in an inositol-requiring mutant of *Neurospora crassa*. J. Bacteriol..

[45] Costa E.A., Subramanian K., Nunnari J., Weissman J.S. (2018). Defining the physiological role of SRP in protein-targeting efficiency and specificity. Science.

[46] Reyes C.L., Rutenber E., Walter P., Stroud R.M. (2007). X-ray structures of the signal recognition particle receptor reveal targeting cycle intermediates. PLoS ONE.

[47] Römisch K., Miller F.W., Dobberstein B., High S. (2006). Human autoantibodies against the 54 kDa protein of the signal recognition particle block function at multiple stages. Arthritis Res. Ther..

[48] Miller J.D., Wilhelm H., Gierasch L., Gilmore R., Walter P. (1993). GTP binding and hydrolysis by the signal recognition particle during initiation of protein translocation. Nature.

[49] Shan S.O., Walter P. (2005). Molecular crosstalk between the nucleotide specificity determinant of the SRP GTPase and the SRP receptor. Biochemistry.

[50] Babst M., Wendland B., Estepa E.J., Emr S.D. (1998). The Vps4p AAA ATPase regulates membrane association of a Vps protein complex required for normal endosome function. EMBO J..

[51] Valko M.M.H.C.M., Morris H., Cronin M.T.D. (2005). Metals, toxicity and oxidative stress. Curr. Med. Chem..

[52] Chaouch S., Noctor G. (2010). Myo-inositol abolishes salicylic acid-dependent cell death and pathogen defence responses triggered by peroxisomal hydrogen peroxide. New Phytol..

[53] Kaye Y., Golani Y., Singer Y., Leshem Y., Cohen G., Ercetin M., Gillaspy G., Levine A. (2011). Inositol polyphosphate 5-phosphatase7 regulates the production of reactive oxygen species and salt tolerance in *Arabidopsis*. Plant Physiol..

[54] Kilaparty S.P., Agarwal R., Singh P., Kannan K., Ali N. (2016). Endoplasmic reticulum stress-induced apoptosis accompanies enhanced expression of multiple inositol polyphosphate phosphatase 1 (Minpp1): A possible role for Minpp1 in cellular stress response. Cell Stress Chaperones.

[55] Saiardi A., Resnick A.C., Snowman A.M., Wendland B., Snyder S.H. (2005). Inositol pyrophosphates regulate cell death and telomere length through phosphoinositide 3-kinase-related protein kinases. Proc. Natl. Acad. Sci. USA.

[56] Zhang X., Shao J., Chen A., Shang C., Hu X., Luo S., Lei M., Peng L., Zeng Q. (2018). Effects of cadmium on calcium homeostasis in the white-rot fungus *Phanerochaete chrysosporium*. Ecotoxicol. Environ. Safety.

[57] Berridge M.J. (1989). Inositol trisphosphate, calcium, lithium, and cell signaling. JAMA.

[58] Huang D., Gong X., Liu Y., Zeng G., Lai C., Bashir H., Zhou L., Wang D., Xu P., Cheng M. (2017). Effects of calcium at toxic concentrations of cadmium in plants. Planta.

[59] Sanderfoot A.A., Raikhel N.V. (1999). The specificity of vesicle trafficking: Coat proteins and SNAREs. Plant Cell.

[60] Brunsch M., Schubert D., Gube M., Ring C., Hanisch L., Linde J., Krause K., Kothe E. (2015). Dynein heavy chain, encoded by two genes in agaricomycetes, is required for nuclear migration in *Schizophyllum commune*. PLoS ONE.

[61] Eitzen G. (2003). Actin remodeling to facilitate membrane fusion. Biochim. Biophys. Acta Mol. Cell Res..

[62] Lanzetti L. (2007). Actin in membrane trafficking. Curr. Opin. Cell Biol..

[63] Nebenführ A., Ritzenthaler C., Robinson D.G. (2002). Brefeldin A: Deciphering an enigmatic inhibitor of secretion. Plant Physiol..

