# Supplementary material for: Inositol Signaling in the Basidiomycete Fungus Schizophyllum commune
Source: J Fungi (Basel). 2021 Jun 10;7(6):470. doi: 10.3390/jof7060470 (PMC8230515; doi:10.3390/jof7060470)
Supplement: Supplementary file 1 [file jof-07-00470-s001.zip › jof-1248003-supplementary.pdf]

Supplemental Material

Suppl. Tab. S1: *S. commune* strains used in this study.

Strain	Genetic background	Source
**12-43**	*ura*$^{-}$	Jena Microbial Resource Collection (JMRC), Germany
**T33**	*matA3,1; matB3,2; trp*$^{-}$*; ura*$^{-}$	JMRC
**EVC1**	*matA3,1; matB3,2*	JMRC
**OEIMP4**	*matA3,1; matB3,2; tef1p::imp1*	JMRC
**OEIMP6**	*matA3,1; matB3,2; tefp::imp1*	JMRC

Suppl. Tab. S2: Primers used in this study.

Name	Gene	Sequence (5'-3')	Length (bp)	Efficiency
**p1for**	pRS415::*ptef1*	*CTAGTTCTAGAGCGGCCGCCACCGCC*GGAAAAGAACAAGACGTGT*		
**p1rev**	*tef1::imp*	*TAGTCGGCGATGGTAAGGTCGGT*GGGCATTTTGAGTGTTTTCTAAGTGAG*		
**p2for**	*tef1::imp*	*TCACTTAGAAAACACTCAAA* ATGCCCACCGACCTTACCAT*		
**p2rev**	*Imp*::pRS415	*CTAAAGGGAACAAAAGCTGG*GACACACGAGGATGACGGTT*		
**mio3**	Myo-inositol oxygenase	For: GGATCTACAAGCCGCACTGT<br>Rev: CACGGTGCCAGGGATAGAAG	149	92.80%
**imp**	Inositol monophos-phatase	For: GAAGCCCGTGCTCGGTG<br>Rev: TGGTTGTTGATGCCCTCCGT		
**ipp1**	Hypothetical inositol polyphosphate phosphatase	For: GACCGCATAGCGGACTACAAC<br>Rev: TCCATGAGGGAATAGCCCGA	188	109.95%
**act**	Actin-1	For: CTGCTCTTGTTATTGACAATGGTTCC<br>Rev: AGGATACCACGCTTGGACTGAGC	178	96.31%
**tef**	Translation elongation factor 1a	For: AGCTCGGCAAGGGTTCCTTCA<br>Rev: AACTTCCAGAGGGCGATATCA	97	97%
**ubi**	Ubiquitin-conjugating-protein	For: GAAGGAGTACGATGCGAAGG<br>Rev: TCCTCCTCTGCCTTCTTGC	93	89.5%

***** *Overhang for annealing in italics*

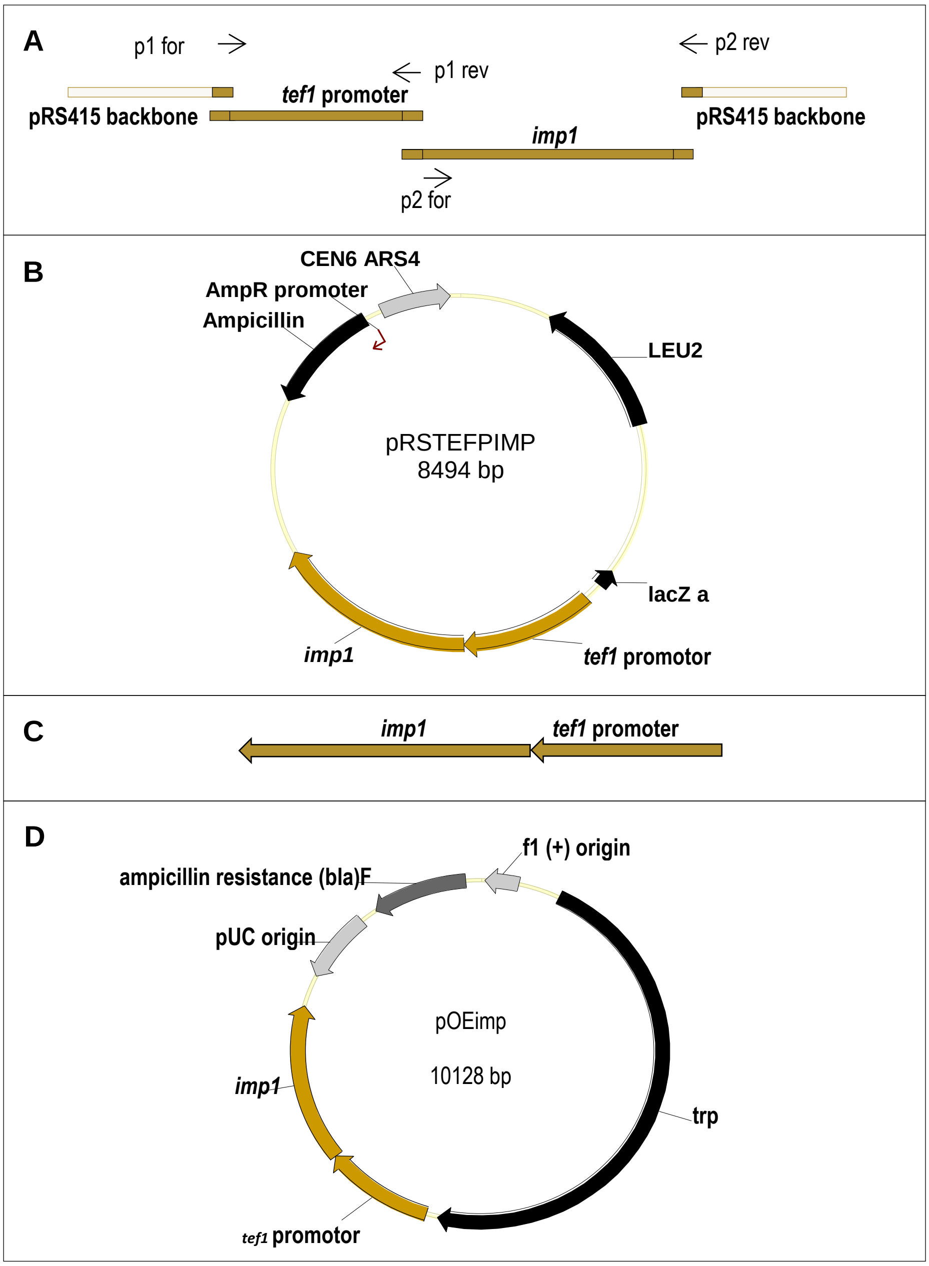

Suppl. Fig. S1: Plasmid construction for generation of *imp1* overexpression strains. (A) Recombination in *S. cerevisiae* was carried out using pRS415 to generate the product fusing the PCR products for the *tef1* promoter (*tef1p*) and the *imp1* gene. (B) Overlapping sequences between two adjacent fragments *tef1p* and *imp1* were used (p1rev and p2for). (C) Fusion product *tef1p::imp1* was cloned in pRS415 to generate pRSTEFPIMP. (D) The *tef1p::imp1* region was excised and cloned into pSK containing the selection marker *trp1* to arrive at pOEIMP.

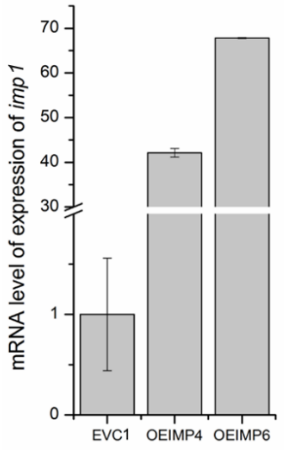

Suppl. Fig. S2: Verification of *imp1* overexpression by RT-qPCR. Comparison is given for the empty vector control EVC1 and overexpression transformants OEIMP4 and OEIMP6.

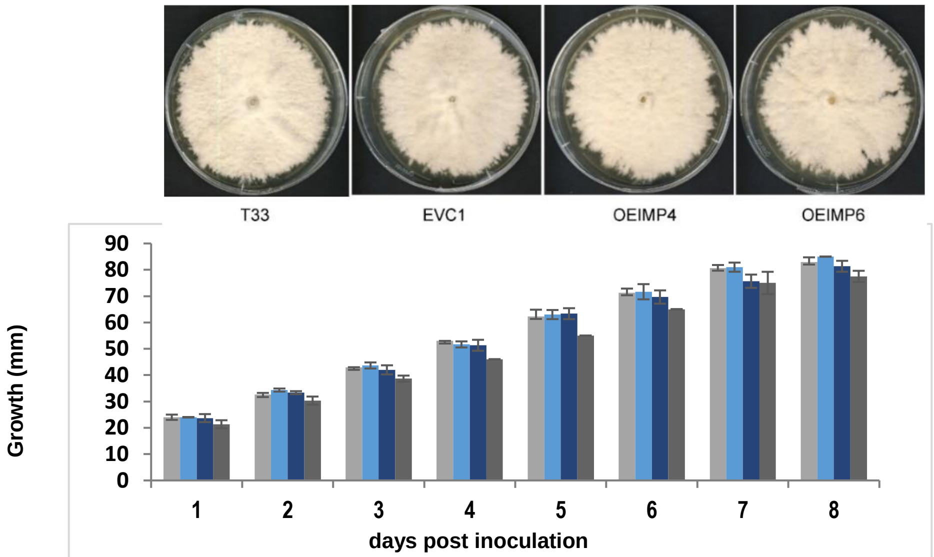

Suppl. Fig. S3: Effect of *imp1* overexpression on colony morphology (top) and growth (below) of *S. commune* T33 (grey bars), empty vector control EVC1 (blue), and overexpression transformants OEIMP4 (dark blue) and OEIMP6 (dark grey); n=3.

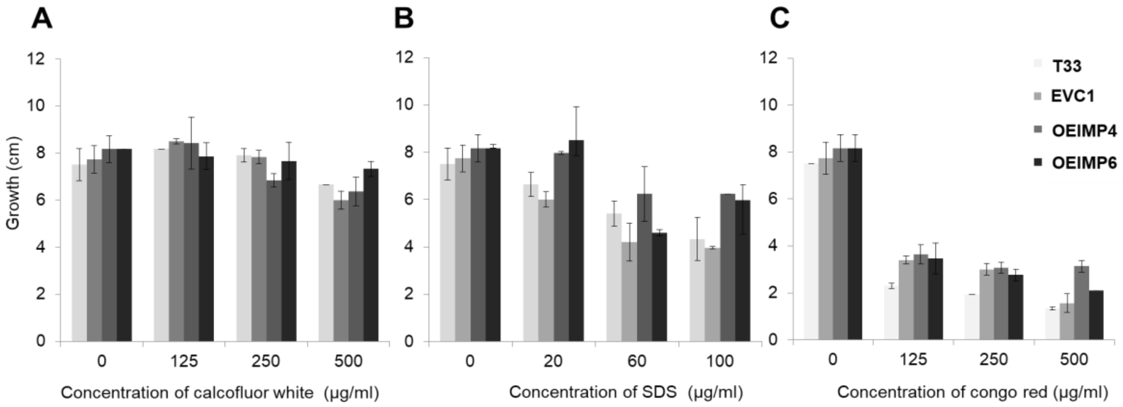

Suppl. Fig. S4: Susceptibility towards calcofluor white (A), SDS (B), and Congo red (C). Comparison is given in triplicates for the wildtype parental *S. commune* T33, the empty vector control EVC1, and overexpression transformants OEIMP4 and OEIMP6.

Suppl. Tab. S3: Regulation of genes involved in the inositol signalling cycle. The microarray analysis (Erdmann et al., 2012) is showing comparisons between *S. commune* 12-43 grown on CYM *versus* CYM with seepage water from a former mining site (HSW) and co-isogenic *S. commune* W22 grown on CYM with 0.01 mM cadmium nitrate.

	KOG Annotation	Gene Location Scho1	12-43 vs. 12-43 HSW			12-43 vs. W22 Cd		
			x-fold	p-value	Q	x-fold	p-value	Q
**PIS**	KOG3240: PI synthase	estExt_fgenesh1_pm.C_20467	-1.45	1.79E-01	1	2.11	1.16E-02	1
	KOG0693: Myo-inositol-1-phosphate synthase	estExt_fgenesh1_pm.C_70303	1.25	4.55E-01	1	2.45	6.76E-03	1
		estExt_fgenesh1_pm.C_70303	2.00	2.81E-03	1	-2.50	2.52E-04	1
		estExt_fgenesh1_pm.C_70303	1.08	6.86E-01	1	-1.50	3.75E-02	1
**PI$_4$K**	KOG2381: PI4-kinase	augustus-scaffold_8.g641	2.24	1.21E-01	1	4.61	6.59E-03	1
		e_gw1.2.405.1	1.25	4.86E-01	1	2.81	3.84E-03	1
	KOG0903: PI4-kinase, involved in intracellular trafficking and secretion,	estExt_Genewise1Plus.C_21667	3.82	9.87E-02	0	1.73	4.86E-01	0
**PI$_5$K**	KOG0230: PI-4-phosphate 5-kinase and related FYVE finger-containing proteins	estExt_fgenesh1_kg.C_60138	-1.75	6.41E-02	1	1.07	8.11E-01	1
		estExt_Genewise1.C_12180	1.04	8.60E-01	1	3.04	1.67E-04	1
	KOG0229: PI-4-phosphate 5-kinase	estExt_fgenesh1_kg.C_110031	2.41	1.38E-02	1	1.75	9.89E-02	1
**PI$_3$K**	KOG0906: PI3-kinase VPS34, involved in signal transduction,	fgenesh1_pm.C_scaffold_500027 2	-1.08	8.16E-01	1	1.34	3.56E-01	1
**PLC**	KOG0169: PI-specific phospholipase C	estExt_Genewise1.C_170034	1.30	2.09E-01	1	-1.06	7.71E-01	1
		estExt_fgenesh1_pm.C_60287	1.18	4.20E-01	1	1.83	9.07E-03	1
		augustus-scaffold_6.g78	-1.81	2.19E-01	1	-1.00	1.00E+00	1
		augustus-scaffold_3.g897	-4.17	5.43E-02	1	2.59	1.87E-01	0
**PKC**	KOG2397: Protein kinase C substrate, 80 KD protein, heavy chain	estExt_Genewise1Plus.C_11897	-1.11	7.10E-01	1	2.56	4.35E-03	1
**IMK**	KOG1620: Inositol polyphosphate multikinase, component of the ARGR transcription regulatory complex,	e_gw1.1.1005.1	1.79	5.90E-01	1	6.94	8.66E-02	1
		estExt_Genewise1Plus.C_10042 7	-15.82	5.24E-03	1	-2.71	2.63E-01	1
	KOG4749: Inositol polyphosphate kinase	e_gw1.2.893.1	-13.45	2.05E-03	1	-6.98	1.46E-02	1
**IP$_3$P**	KOG1089: Myotubularin-related PI3-phosphate 3-phosphatase MTM6	estExt_fgenesh1_pm.C_20581	3.88	2.81E-02	1	2.11	2.04E-01	1
**IP$_4$P**								
**IP$_5$P**	KOG0565: Inositol polyphosphate 5-phosphatase and related proteins	estExt_fgenesh1_pm.C_20547	1.35	7.50E-01	1	7.38	4.55E-02	1
		estExt_Genewise1.C_170073	-1.20	4.45E-01	1	1.13	6.01E-01	1
		augustus-scaffold_5.g421	-9.43	1.26E-01	1	-5.25	2.51E-01	1
		estExt_fgenesh2_pg.C_100071	-8.56	1.58E-04	1	-12.9	2.38E-05	1
**IPP**	KOG1382: Multiple inositol polyphosphate phosphatase	gw1.1.3820.1	1.86	1.58E-01	1	1.96	1.29E-01	1
		gw1.8.732.1	1.55	2.01E-01	1	2.12	3.59E-02	1
		estExt_Genewise1Plus.C_30697	1.35	4.05E-01	1	-2.21	3.69E-02	1
		e_gw1.7.1022.1	-1.62	5.16E-01	1	-1.10	9.02E-01	1

	KOG Annotation	Gene Location Scho1	12-43 vs. 12-43 HSW			12-43 vs. W22 Cd		
			x-fold	p-value	Q	x-fold	p-value	Q
ITP	KOG0566: Inositol-1,4,5-triphosphate 5-phosphatase (synaptojanin), INP51/INP52/INP53 family	estExt_Genewise1.C_11249	-9.00	4.61E-02	1	-14.53	1.79E-02	1
IMP	KOG2951: Inositol monophosphatase	e_gw1.1.586.1	1.33	4.63E-01	1	1.12	7.67E-01	1
		e_gw1.1.586.1	-1.05	8.93E-01	1	-1.48	2.59E-01	1
		e_gw1.1.586.1	-1.30	2.04E-01	1	-1.20	3.79E-01	1

Suppl. Tab. S4: Intracellular trafficking associated proteins regulated in *imp1* overexpressing transformant pOEIMP4 vs. EVC

ID	x-fold	KOG class and description		BLAST hit, organism, % identity
2598708	2.9	cyto-skeleton	Drebrins and related actin binding proteins	Actin-binding related protein, *Laccaria bicolor*, 70%
2541047	2.7		Kinesin-like protein	Kinesin-like protein, *Armillaria solidipes*, 78%
2674409	2.3		Predicted actin-bundling protein	Protein FRG1, *Hypsizygus marmoreus*, 68%
2681330	2.3		Predicted actin-bundling protein	Ubiquitin-activating enzyme E1-like, *Trametes pubescens*, 63%
2704529	23.8	intra-cellular traf-ficking, secretion, and vesicular transport	Signal recognition particle, subunit Srp19	Signal recognition particle, SRP19 subunit, *Armillaria solidipes,* 61%
2262522	23.7		Protein involved in glucose derepression and pre-vacuolar endosome protein sorting	Snf7-domain-containing protein, *Armillaria solidipes*, 74%
2603193	21.7		Mitochondrial Fe/S cluster exporter, ABC superfamily	Iron-sulfur clusters transporter ATM1, *Coprinopsis cinerea okayama,* 75%
2597192	21.7		Golgi transport complex subunit	Dor1-domain-containing protein, *Armillaria gallica*, 48%
2678633	4.3		Vesicle coat complex AP-3, beta subunit	acyltransferase ChoActase/COT/CP, *Fistulina hepatica*, 60%
2693248	3.5		Signal recognition particle, subunit Srp72	Signal recognition particle subunit SRP72, *Trametes pubescens*, 47%
2460397	3.0		Mitochondrial import inner membrane translocase, subunit TIM8	Mitochondrial import inner membrane translocase *Heterobasidion irregulare*, 68%
2700443	3.0		Guanine nucleotide exchange factor	Sec7 guanine nucleotide exchange factor, *Moniliophthora roreri*, 72%
1185145	2.4		Vacuolar assembly/sorting protein DID2	Vacuolar protein sorting protein 46, *Moniliophthora roreri*, 90%
2698910	2.2		Membrane coat complex Retromer, subunit VPS5/SNX1, Sorting nexins, and related PX domain-containing proteins	PX-domain-containing protein, *Armillaria gallica*, 75%<br>Sorting nexin-41, Hypsizygus marmoreus, 76%
2678145	2.1		Cysteine protease required for autophagy - Apg4p/Aut2p	Thiamine pyrophosphokinase, *Coprinopsis cinerea okayama*, 49%
2007874	2.1		Nuclear transport factor 2	Nuclear transport factor 2, *Neolentinus lepideus*, 75%
2595664	2.1		SNARE protein Syntaxin 1 and related proteins	t-SNARE, *Armillaria solidipes*, 80%
2573839	2.2	poorly characte-rized	Vacuolar sorting protein VPS1, dynamin, and related proteins	Dynamin protein dnm1, *Moniliophthora roreri MCA 2997*, 75%

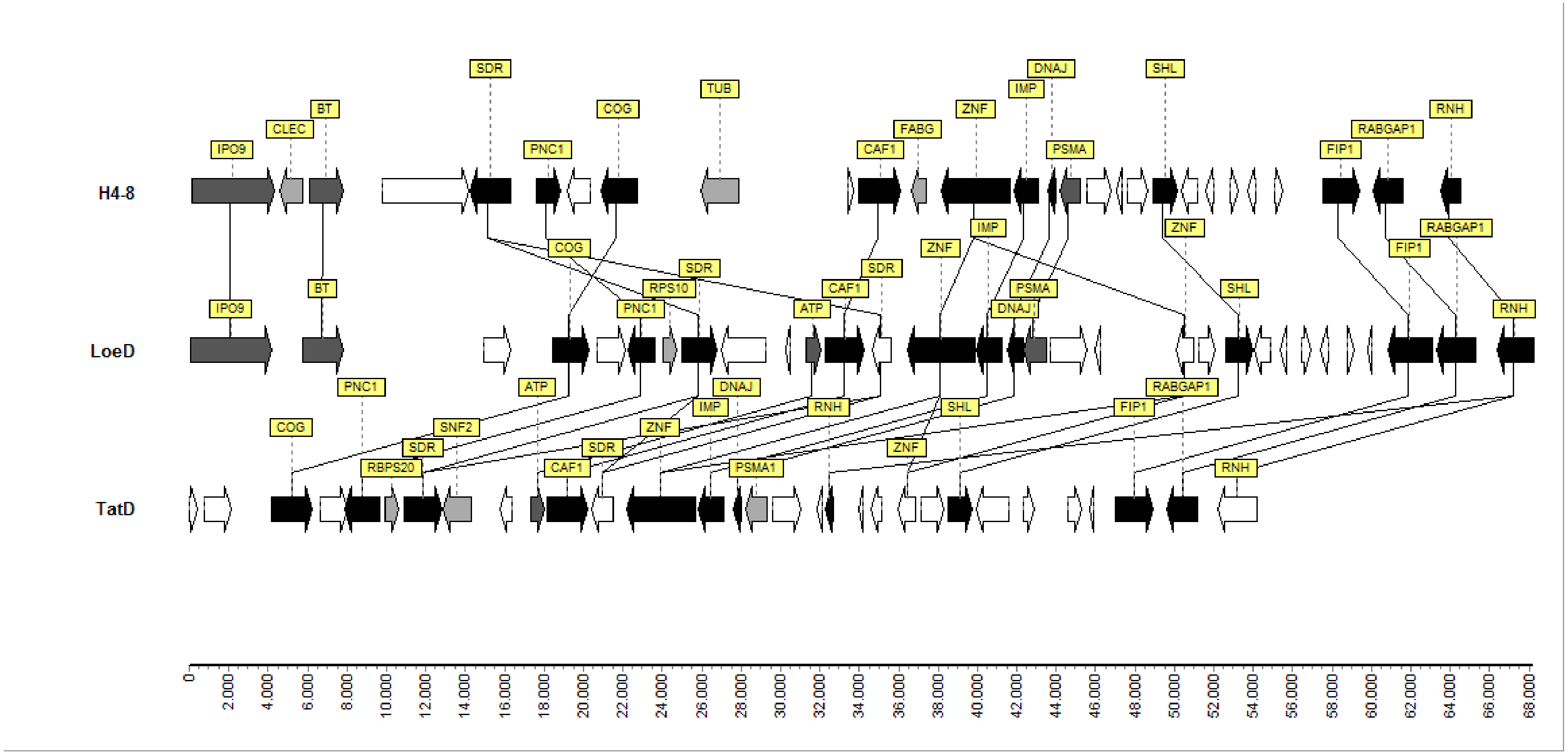

Suppl. Fig. S5: Chromosomal map of *imp1* surrounding genes in three different strains *S. commune,* H4-8, LoeD, and TatD. White arrows indicate unknown genes, black arrows show that the corresponding genes are present in all three genomes.
